# Colchicine: A Dual Therapeutic Target for Trichinellosis

**DOI:** 10.1007/s11686-024-00979-9

**Published:** 2025-01-24

**Authors:** Enas Fakhry Abdel Hamed, Afaf A. Taha, Shereen M. Abdel Ghany, Al-Sayed R. Al-Attar, Eman M. Fawzy

**Affiliations:** 1 Department of Medical Parasitology, Faculty of Medicine, Zagazig University, El Kawmia Square, Zagazig, Sharkia Governorate Egypt; 2 Department of Pathology, Faculty of Veterinary Medicine, Zagazig University, Sharkia, Egypt

**Keywords:** Trichinellosis, Colchicine, Acetazolamide, Atorvastatin

## Abstract

**Purpose:**

Trichinellosis affects around 11 million people globally. Treatments for this medical condition are limited by adverse effects and resistance, emphasising the importance of effective and safe therapies. Consequentially, we sought to study colchicine’s synergistic effects with atorvastatin or acetazolamide in the treatment of *Trichinella spiralis* (*T. spiralis)*-infected mice.

**Methods:**

Seventy mice were evenly divided into two groups (a and b) of 35 each. During the intestinal phase, group (a) began therapy on the second day post-infection (dpi) and lasted four days. Group (b) had treatment for four weeks during the muscle phase, beginning on the 12th dpi. While the other five infected groups received atorvastatin, colchicine, acetazolamide, a combination of acetazolamide and colchicine, or none, one group of infected mice received no treatment at all as a negative control. The efficacy was assessed by parasite count, histopathology and scanning electron microscopy.

**Results:**

Our data revealed that the combination treatment lowered *T. spiralis* adult worm and larvae counts in infected animals. Moreover, it restored the normal intestinal and muscular architecture, reduced edema, and alleviated inflammation, as demonstrated by reduced inflammatory infiltrate. Scanning electron microscopic examination of adults and larvae verified our findings.

**Conclusion:**

Adjuvant treatment with colchicine as an antifibrotic can help treat muscle trichinellosis by reducing the production of fibrous tissue. This might help to enhance treatment results by enabling the admission of larvicidal medications and, as a result, reducing the number of larvae in the muscle, which together form the basis of pathology and can be debilitating for the patient.

## Introduction

Trichinellosis is recognized as a worldwide zoonosis causedby nematodes belonging to the genus *Trichinella* [1]. It is one of the most prevalent neglected tropical illnesses in the world, causing chronic infection in a wide range of humans and animals. Human patients become infected after ingesting raw or undercooked pork containing infective *Trichinella* larvae [2]. There are three stages of human trichinellosis: the intestinal phase, the migratory phase, and the muscular phase. The parasite pierces host muscle cells, inducing muscle inflammation. For the most part, encapsulated muscle larvae cause morbidity. These larvae create a pathogenesis that is aggressive and ultimately results in neurological and cardiac conditions that are deadly [3].

Albendazole (ALB) and mebendazole are the traditional drugs used to treat trichinellosis. However, their poor water solubility restricts drug absorption. Moreover, prolonged, high-dose therapy may result in reversible elevation of aminotransferase level, bone marrow suppression, and alopecia [4]. Therefore, finding a safe and efficient medication is mandatory [5].

Acetazolamide is a sulfonamide that eliminates altitude sickness, idiopathic intracranial hypertension, and epileptic seizures by inhibiting carbonic anhydrase [6]. Furthermore, as shown by Giacomotto et al. [7], carbonic anhydrase inhibitors have some benefits for improving degenerating muscle. Beta carbonic anhydrases (β-CA), mitochondrial enzymes, have been detected in many species of nematodes including *T. spiralis*. Parasite genomes have β-CA genes, but vertebrate genomes do not. One of the CA inhibitors that works against *T. spiralis* is acetazolamide, which can interact with the mitochondrial metabolic cycles and kill the parasite [8]. Additionally, Abdel Hamed et al. [9] observed that treatment with acetazolamide led to the degeneration of the larvae and capsule, suggesting a potential therapeutic role for trichinellosis.

Araújo et al. [10] found that atorvastatin’s biological activity differs from its initial therapeutic intent. Statins are used to treat hypolipidemia and have a variety of benefits, including suppressing inflammation [11], inhibiting cellular proliferation [12], and improving endothelial function. Since angiogenesis is very important for the existence of the *T. spiralis* nurse cell complex, inhibition of angiogenesis by reduction of vascular endothelial cell growth factor (VEGF) level by atorvastatin may be one of the suggested mechanisms for suppression of *T. spiralis* larval growth [10]. Furthermore, atorvastatin has been used in experimental *T. spiralis* infections to suppress angiogenesis and reduce inflammation [13].

Colchicine is an alkaloid anti-mitotic agent used to treat inflammation and fibrosis [14]. Its pharmacological effects are related to inhibiting microtubule self-assembly by binding to tubulin, limiting intercellular granule mobility and chemical release [15]. It is used to treat familial Mediterranean fever (FMF) [16], chronic inflammatory disorders such as gouty arthritis [14], and fibro-inflammatory diseases [17], as well as COVID-19 [18]. Antifibrotic medications may be used as adjuvant therapy in conjunction with antiparasitic medications to improve therapeutic outcomes in muscular trichinellosis [19]. ElGhannam et al. [20] developed a unique therapy for both stages of trichinellosis using eugenol’s anti-inflammatory capabilities. Nikolaidis et al. [21] reported that colchicine can interfere with nurse cell collagen synthesis by *T. spirlais*-encysted larvae in muscles by indirectly altering the expression of various genes, including the fibronectin gene. Based on the criteria mentioned above, this study sought to assess the synergistic effect of colchicine as an adjuvant medication to either atorvastatin or acetazolamide in treating *T. spiralis*-infected mice.

## Materials and Methods

### Ethical Considerations

This study was approved by the ethical committee of ZagazigUniversity, the Faculty of Medicine, and the Institutional Animal Care and Use Committee ((ZU-IACUC. 3/F/57/2020).All studies at Theodor Bilharz Research Institute (TBRI) were conducted in compliance with the Clinical and Laboratory Standards Institute (CLSI) criteria and authorized by the institution for animal ethics in terms of animal care and waste disposal.

### Experimental Animals and Infection

Seventy male Swiss albino mice, aged 6–8 weeks and weighing 25–30 g, were raised in a well-organized TBRI laboratory setting with a temperature of 22 °C, a regulated light/dark cycle, and 56% humidity. The *T. spiralis* strain was supplied by the TBRI. The infectious larvae were obtained by digesting infected mouse diaphragms in 1% pepsin-hydrochloride, then incubated at 37 °C overnight, and then washed in physiological saline (0.85%) four times. The larvae were collected using the sedimentation method. They were used to infect mice orally with 200–250 larvae each [22].

### Drug Dosage Schedule

To give mice the medications, acetazolamide 250 mg tablets were dissolved in distilled water and given orally at 100 mg/kg/day as recommended by Saad et al. [23], atorvastatin 20 mg tablets were dissolved in distilled water and given orally at 10 mg/kg/day [13], and colchicine 500 µg tablets were dissolved in distilled water and given by oral gavage daily at a dose of 200 µg/kg/day [19].

### Sampling Methodology and Experimental Design

Seventy mice were allocated equally into two groups (a and b) of 35 each. During the intestinal phase, group (a) started therapy on the subsequent day post-infection (dpi) and lasted four days. Group (b) had treatment for four weeks during the muscle phase, beginning on the 12th dpi. Both groups (a and b) were subdivided into seven subgroups of five mice each: Group I was the negative control, Group II was the positive infected control, and Group ΙΙΙ infected mice were treated with a 200 µg/kg single oral dose (SOD) of colchicine. Group IV-infected mice were treated with 100 mg/kg SOD of acetazolamide. Group V-infected mice were treated with a combination of 100 µg/kg acetazolamide and 200 µg/kg colchicine. Group VΙ-infected mice were treated with 10 mg/kg of atorvastatin SOD, and group VII-infected mice were treated with a combination of 10 mg/kg atorvastatin and 200 µg/kg colchicine. On the sixth day dpi, mice from group (a) were sacrificed. On the 42nd dpi, mice from group (b) were sacrificed.

### Parasitological Study

#### Mortality Rates (MR %)

The following equation was used to determine the death rate of the experimental subgroups at the moment of sacrifice: MR% is calculated as follows: total number of mice at trial start × 100 / number of dead mice at sacrifice time [24].

#### Adult Worm Counting in the Intestines (Mean Number of Living Adults/Mouse)

The intestinal canal was sliced lengthwise and incubated in 10 ml of saline for 2 h at 37 °C after being cropped into 1 cm pieces. The intestine was then gently scraped to gather the lodged adults in the mucosa. The fluid had been washed many times until it cleared. The fluid was then centrifuged for five minutes at 1500 rpm. After removal of the supernatant, the sediment containing adults was observed by a microscope for counting, following Wakelin and Lloyd’s procedure [25].

#### Calculating the Overall Larval Load in the Muscles (Average Number of Larvae Per Mouse)

First, a compression diagnostic approach was employed to confirm infection and the presence of *T. spiralis* larvae. A tiny layer of skeletal muscle from each mouse was squeezed between two slides and examined using a low-power objective [26]. Each mouse carcass was artificially digested for two hours in distilled water containing 1% pepsin and 1% HCl. The muscle larval numbers were determined in a manner similar to the prior approach of preparing muscle larvae for infection [22].

The effectiveness of every medicine was determined using the equation. Efficacy% is calculated as (A-B) A x 100, where A is the number of worms or larvae removed from infected, untreated mice (positive control) and B is the number of worms or larvae removed from each group.

#### Histopathological Examination

From mice sacrificed at 6 dpi, a centimetre of jejunum at the point where the proximal 1/3 and distal 2/3 meet was collected, while lingual and skeletal muscle specimens were obtained from animals sacrificed at 42 dpi. After being cleansed with xylol, the specimens were dehydrated in progressively higher grades of ethanol and inserted in pure soft paraffin blocks after being settled for 24 h in 10% neutral-buffered formalin. Microtome slices of 5 μm thickness were hematoxylin and eosin (H&E) as described by Carleton et al. [27].

### Scanning Electron Microscopy

Worms and larvae were transferred to a fixation solution (pH 7.2) containing 2.5% glutaraldehyde (w/v) in 0.1 M sodium cacodylate, and the solution was maintained at 3 °C overnight. Worms and larvae were washed at pH 7.2 in 0.1 M sodium cacodylate buffer for five minutes before being fixed for one hour in osmium tetroxide 2% (w/v) in sodium cacodylate buffer. The samples were placed on a counterfoil after being dried with liquid carbon dioxide and dehydrated using an alcohol series. Following their gold-suspended preparation, they were examined using Jeol scanning electron microscope (Jeol Corp., Mitaka, Japan) [28].

### Statistical Analysis

The measured parameters’ quantitative values were presented as mean ± standard deviation (SD). Using the Statistical Package for Social Sciences (SPSS), version 26, the data were analyzedusing one-way ANOVA with the Tukey post hoc test to ascertain the significance of differences between groups. When *P* < 0.05, the difference was deemed statistically significant, and when *P* < 0.01, it was deemed extremely significant.

## Results

### The in Vivo Effects of Colchicine Combined with Either Acetazolamide or Atorvastatin Against Adults and Muscle Larvae

Table (1a) displays a highly significant difference between the examined groups in terms of *T. spiralis* adult count in the small intestine (F = 170, *P* < 0.001). The mean adult count of *T. spiralis* was significantly lower among GVa and GIVa groups compared to the other groups (24.6, 32.3 versus 87, 77.2, 56.2, and 50.5, respectively). When comparing GVa and GIVa to the other groups, the greatest percentage drop in adult count was seen in these two groups (71.2% and 62.9%, respectively). Furthermore, no deaths were reported.

**Table 1a:** The mean *T. spiralis* adult count in the small intestine of subgroups (a) on 6th day P.I.

Groups	GIIIa colch.	GIVa aceta	GVa Comb.	GVIa atorv.	GVIIa comb.	GIIIa colch.
Mean ± SD	87.00 ± 7.88	77.2 ± 5.51	32.2 ± 4.61	24.6 ± 5.01	56.2 ± 7.53	50.5 ± 3.65
P value	< 0.001**					
F test	170					
Percentage of reduction		11.2%	62.9%	71.2%	35.4%	41.9%
Mortality%	10%	10%	0%	0%	10%	10%

F: One-way ANOVA test. **: highly significant difference

The number of mature t-spiralis counts in the small intestine differed significantly (*p* < 0.001) across subgroups. However, there was no significant difference between GIVa and GVa (*P* = 0.06) or between GVIa and GVIIa (*P* = 0.20) (Table 1b).

**Table 1b:** Comparison of mean *T. spiralis* adult count in the small intestine of subgroups (a) on 6th day between separate groups

Groups	GIIa Infect. Cont.	GIIIa colch.	GIVa aceta	GVa Comb.	GVIa atorv.	GVIIa comb.
GIIa	---	0.01*	< 0.001**	< 0.001**	< 0.001**	< 0.001**
GIIIa	0.01*	---	< 0.001**	< 0.001**	< 0.001**	< 0.001**
GIVa	< 0.001**	< 0.001**	---	0.06	< 0.001**	< 0.001**
GVa	< 0.001**	< 0.001**	0.06	---	< 0.001**	< 0.001**
GVIa	< 0.001**	< 0.001**	< 0.001**	< 0.001**	---	0.20
GVIIa	< 0.001**	< 0.001**	< 0.001**	< 0.001**	0.20	---

Tukey post-hoc test. *: significant difference (*p* < 0.05). **: highly significant difference (*p* < 0.001)

On the 45th day post-intervention, Table 2a demonstrates that there was a highly significant difference (F = 483, *P* < 0.001) in the *T. spiralis* larval count between the examined groups. The GVb and GIVb groups had considerably lower mean larval counts of *T. spiralis* than the other groups (23.2, 39.0 vs. 98.4, 76.2, 65.6, and 55.4, respectively). In comparison to the other groups, GVb and GIVb had the largest percent drop in larval count (75.2% and 60.3%, respectively). Additionally, no deaths were noted in the GVb and GIVb groups.

**Table 2a:** The mean *T. spiralis* encysted larval count in the studied subgroups (b) on the 45th day P.I

Groups	GIIb Infect. Cont.	GIIIb colch.	GIVb aceta	GVb Comb.	GVIb atorv.	GVIIb comb.
Mean ± SD	98.4 ± 3.3	76.2 ± 2.3	39.0 ± 2.7	23.2 ± 1.9	65.6 ± 3.5	55.4 ± 2.0
P value	< 0.001**					
F test	**483**					
Percentage of reduction		22.5%	60.3%	75.2%	33.3%	43.6%
Mortality%	20%	10%	0%	0%	10%	0%

Upon doing a separate comparison of the larval counts of *T. spiralis* between each two groups, a highly significant difference (*p* < 0.001) was seen between the subgroups and one another. But there was a significant difference (*P* = 0.01) between GVIb and GVIIb and between GIVb and GVb (Table 2b).

**Table 2b:** Comparison of mean *T. spiralis* larval count of subgroups (a) on 45th day between separate groups

Groups	GIIb Infect. Cont.	GIIIb colch.	GIVb aceta	GVb Comb.	GVIb atorv.	GVIIb comb.
GIIb	---	0.01*	< 0.001**	< 0.001**	< 0.001**	< 0.001**
GIIIb	0.01*	**---**	< 0.001**	< 0.001**	< 0.001**	< 0.001**
GIVb	< 0.001**	< 0.001**	---	0.01*	< 0.001**	< 0.001**
GVb	< 0.001**	< 0.001**	0.01*	---	< 0.001**	< 0.001**
GVIb	< 0.001**	< 0.001**	< 0.001**	< 0.001**	---	0.01*
GVIIb	< 0.001**	< 0.001**	< 0.001**	< 0.001**	0.01*	---

Tukey post-hoc test. *: significant difference (*p* < 0.05). **: highly significant difference (*p* < 0.001)

### Histopathological Findings

**Fig. 1:**
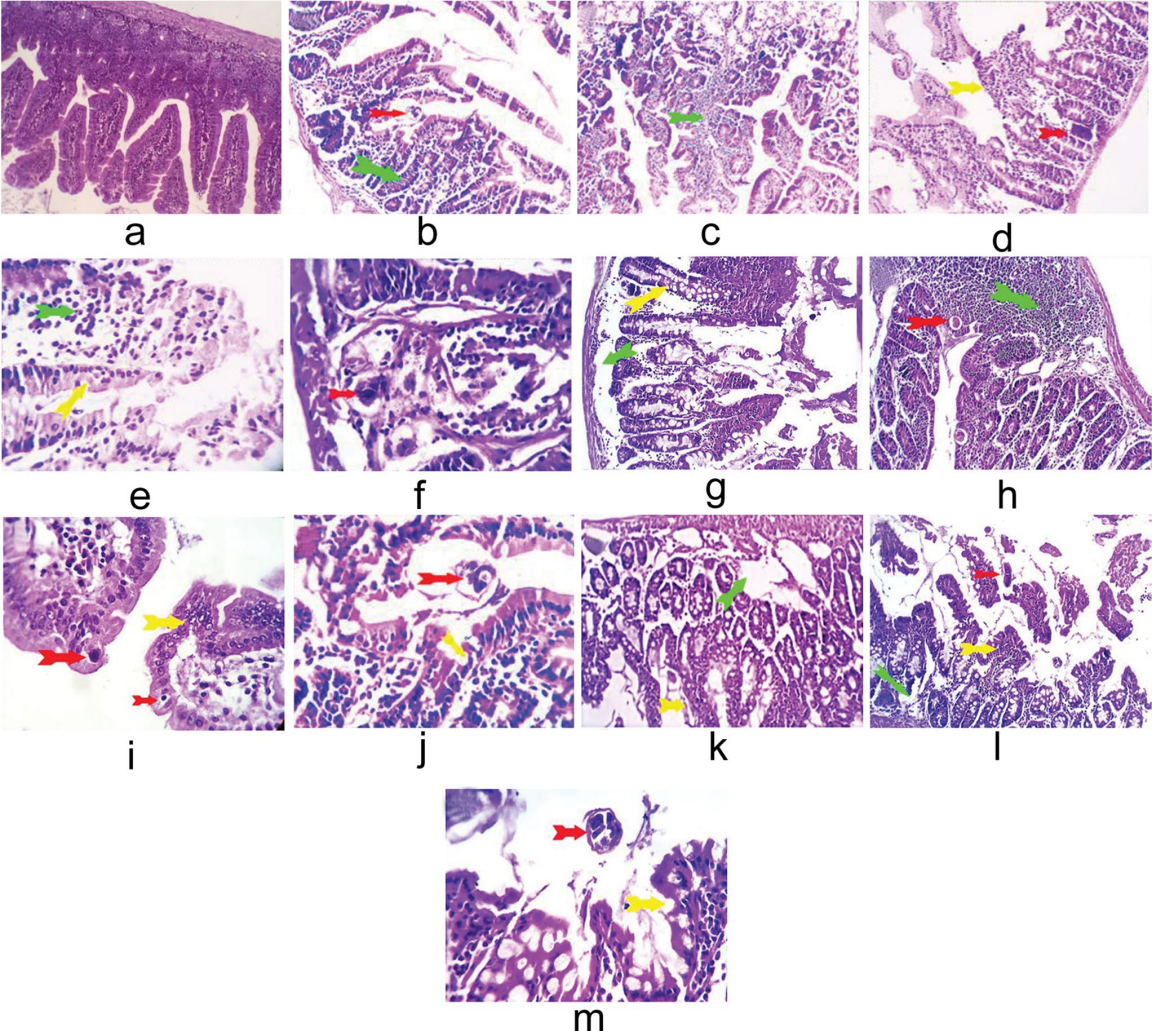
Photomicrographs from the studied groups’ small intestines (X 200&X400). (**a**) GIa with normal histological structures of villi, crypt, glands, mucosa, and muscle layers. (**b**) GIIa with intense invasion by *T. spiralis* adults (red arrows) and pressure atrophy in the cells. The surrounding intestinal mucosa showed mucosal edema and excessive inflammatory reactions, mostly lymphocytes (green arrows). (**d**, **e**) an intestinal segment of GIIIa with *T. spirals* adults (red arrow). The intestinal villi appeared edematous (yellow arrow), and severe mucosal infiltration by lymphocytes and eosinophils was seen (green arrow). (**f**, **g**) GIVa with moderate mucosal invasion by *T. spiralis* adult (red arrows). The intestinal villi were normal in most parts, apart from focal pressure atrophy (yellow arrows). Moderate mucosal and submucosal infiltration by lymphocytes and eosinophils together with partially degenerated intestinal gland and submucosal edema were seen (green arrows). (**h**, **i**) GVa with moderate mucosal invasion by degenerated *Trichinella* adults (red arrows). The intestinal villi were normal in most parts, a part of focal pressure atrophy and/or epithelial stratification (yellow arrows). Mild mucosal and submucosal infiltration by lymphoplasmacytes and hyperplastic ileal Peyer’s patches were seen (green arrows). (**j**,**k**) GVIa with mucosal *Trichinella* adults (red arrows). The intestinal villi showed focal pressure atrophy (yellow arrows). Moderate mucosal and submucosal infiltrations by lymphocytes and eosinophils, together with hyperplastic ileal payer’s patches (green arrows). (**l**,**m**) GVIIa with moderate mucosal invasion by *T. spiralis* adults (red arrows). The intestinal villi were normal in most parts, a part of focal pressure atrophy (yellow arrows). Moderate mucosal and submucosal infiltration by lymphocytes and eosinophils, partially degenerated intestinal glands, and submucosal edema were seen (green arrows)

The best improvement of small intestinal tissue was in GVa, which exhibited mild mucosal invasion by degenerated *T. spiralis* adults and mild mucosal and submucosal infiltration by lymphoplasmacytes with hyperplastic ileal Peyer’s patches (Fig. 1h, i) compared to GIIa, which revealed a severe invasion of the cells by mature *T. spiralis*, together with pressure atrophy, severe mucosal edema, and excessive inflammation in the surrounding intestinal mucosa (Fig. 1b, c). With partly deteriorated intestinal glands and submucosal edoema, both GIVa (Fig. 1f, g) and GVIIa (Fig. 1l, m) showed a medium mucosal and submucosal infiltrate by eosinophils and lymphocytes. On the other hand, GIIIa (Fig. 1d, e) and GVIa (Fig. 1j, k) showed mucosal invasion with *T. spirals* adults in the company of edematous intestinal villi and severe mucosal infiltration by lymphocytes and eosinophils.

**Fig. 2:**
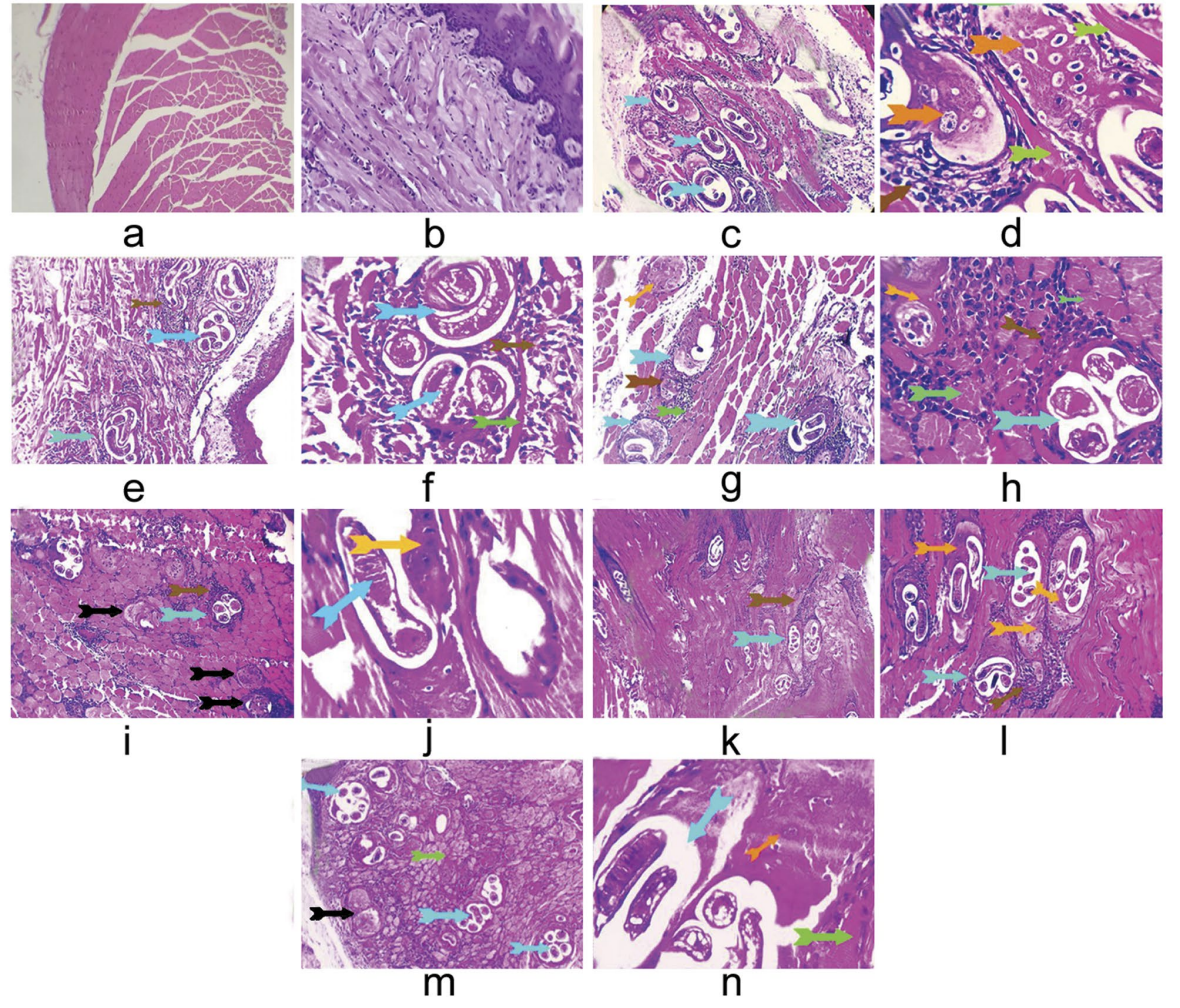
Photomicrographs from the studied groups’ skeletal and lingual muscles (X 200& X400). (**a**,**b**) GIb with normal histological structures of skeletal and lingual muscles, preserved longitudinal cross striations, peripherally situated multinuclear arrangement, interstitial tissue, and tongue papillae. (**c**,**d**) GIIb with excessively encysted *Trichinella* larvae (light blue arrows). They were surrounded by an excessive inflammatory hypersensitivity reaction formed from eosinophils, lymphocytes, and macrophages (brown arrows). A characteristic metaplastic cartilage around some parasitic cysts in some sections (yellow arrows). The surrounding muscle shows degenerative and necrotic changes and interstitial inflammatory edema (green and black arrows). (**e**,**f**) GIIIb shows encysted *Trichinella* larvae with a very thin fibrous capsule (light blue arrows). They were surrounded by severe inflammatory reactions of eosinophils and round cells (brown arrows). The surrounding muscle denoted degenerative and necrotic changes beside interstitial edema (green arrows). (**g**,**h**) GIVb with moderate invasion by encysted *Trichinella* larvae (light blue arrows); a few of them were degenerated and replaced by characteristically transformed mesenchymal tissue (fibrocartilagenous tissue) (orange arrows). They were surrounded by a moderate inflammatory reaction, mostly of round cells (lymphocytes and macrophages) (brown arrows). The surrounding muscle fibers revealed degenerative changes (green arrows). (**i**,**j**) GVb shows a mild invasion by *T. spiralis* larvae with an absent fibrous capsule (light blue arrows) and surrounded by a mild inflammatory hypersensitive reaction formed from eosinophils, lymphocytes, and macrophages (brown arrows). A characteristically transformed mesenchymal tissue was seen around parasitic cysts (yellow arrow) and a large number of degenerated *Trichnella* larvae were replaced by exudate (black arrow). (**k**,**l**) GVIb with moderate invasion by encysted *Trichinella* larvae (light blue arrows); some of them were degenerated and replaced by characteristically transformed mesenchymal tissue (fibrocartilagenous tissue) (orange arrows). They were surrounded by a moderate inflammatory reaction, mostly of round cells (lymphocytes and macrophages) (brown arrows). The surrounding muscle fibers revealed degenerative changes (green arrows). (**m**,**n**) GVIIb shows a moderate invasion by encysted *Trichinella* larvae (light blue arrows) with missing capsules. Most of the larvae were degenerated and replaced by inflammatory or transformed mesenchymal tissue (fibrocartilagenous tissue) (orange arrows). The surrounding muscle showed necrotic changes (green arrows). Some of degenerated *Trichnella* larvae were replaced by exudate (black arrow)

The muscle sections in the targeted GVb (Fig. 2i, j) displayed mild invasion by *T. spiralis* larvae with an absent fibrous capsule and a mild inflammatory hypersensitivity reaction. Furthermore, a decreased number and size of necrotic larval nurse cells that were replaced by exudates were observed (Fig. 2i). Empty larval nurse cells were detected (Fig. 2j) compared to GIIb, which showed excessively encysted *Trichinella* larvae surrounded by an excessive inflammatory hypersensitivity reaction with characteristic metaplastic cartilage around some parasitic cysts. Figure 2c, d depicts interstitial inflammatory edoema together with degenerative and necrotic alterations in the surrounding muscle. GVIIb revealed a moderate invasion by encysted *Trichinella* larvae with missing capsules. With necrotic muscle around them, the majority of the larvae had deteriorated and been replaced by inflammatory or altered mesenchymal tissue (Fig. 2m, n). Despite GIVb (Fig. 2g, h) having moderate invasion by encysted *Trichinella* larvae and GVIb (Fig. 2k, l) having excessive invasion by encysted *Trichinella* larvae, a moderate inflammatory response surrounded some of the degenerating larvae that were replaced by mesenchymal tissue. The surrounding muscle fibers revealed degenerative changes. Conversely, GIIIb (Fig. 2e, f) shows encysted *Trichinella* larvae with a very thin fibrous capsule surrounded by severe inflammatory reactions. Interstitial edoema and degenerative and necrotic alterations were evident in the surrounding muscle.

### Scanning Electron Microscopy

**Fig. 3:**
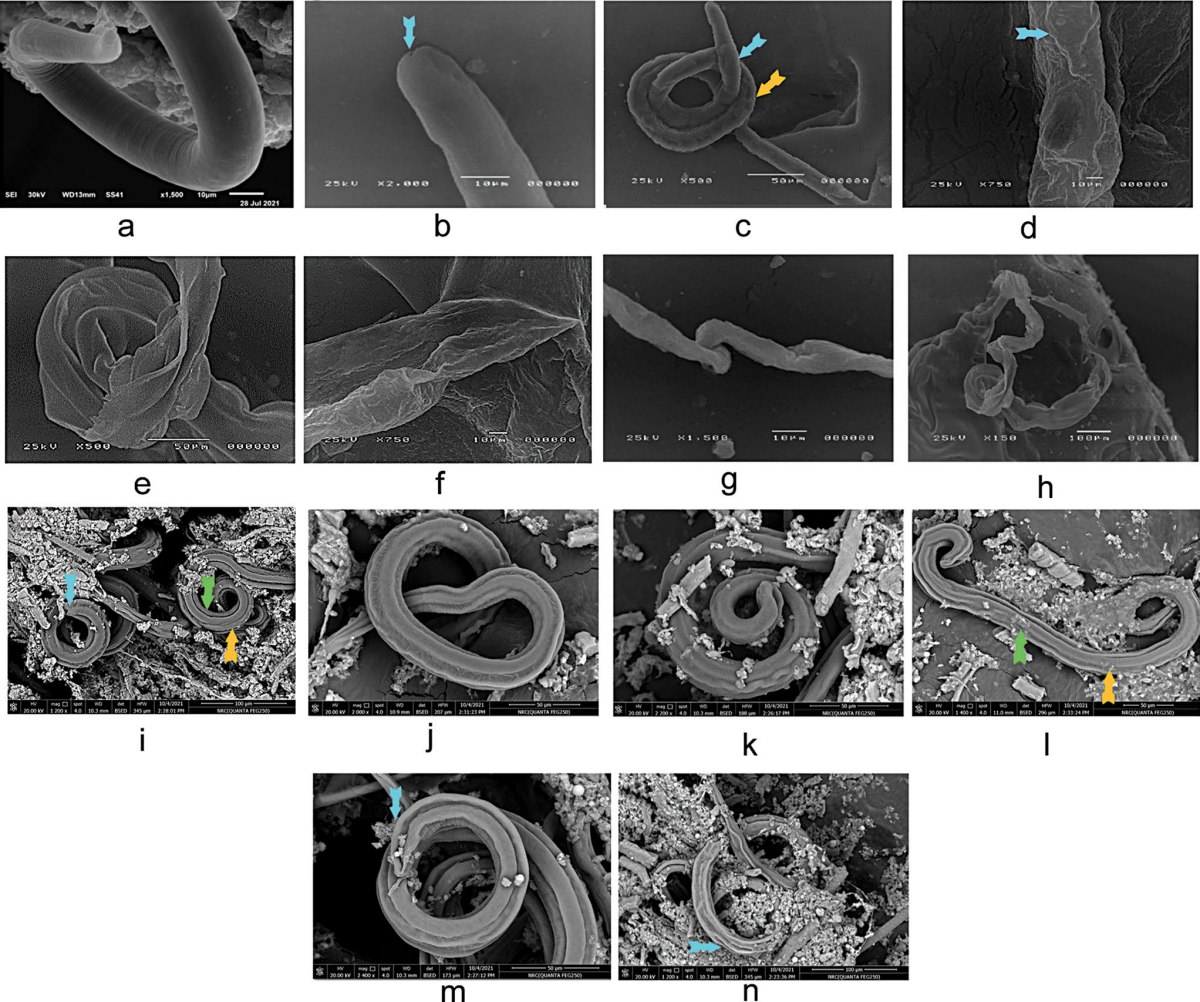
Scanning electron micrography of adult and larval *T.spiralis*. (**a**,**b**) A normal *T.spiralis* adult normal rounded posterior end, anus appears at the tip of the posterior end (blue arrow). (**c**) GIIIa presents a cauliflower mass on the body (blue arrow) with multiple depressions (yellow arrow). Intact smooth non wrinkled epidermis. (**d**) GIVa with loss of transverse striations with blebs (blue arrow). Mild focal of wall epidermis with a mild degree of carrions. (**e**,**f**) GVa with loss of transverse striations along the body with severely collapsed body in (**e**) and damaged longitudinal ridges with fissures with flattened annulations at the posterior end in (**f**). (**g**) GVIa shows disfigurement of the cuticle with multiple depressions and the disappearance of intact annuli with corrugation and shrinking of the surface epidermal layer. (**h**) GVIIa with a severely collapsed body and a sloughed cuticle. Serious alterations in the worm cuticle that was severely damaged and detachment of the epidermis and the sub-dermal layer which appear irregularly arranged. (**i**) A normal *T. spiralis* larval with a normal coiled appearance. A normal annulation at the posterior end (blue arrow) with normal transverse creases (green arrow) along the body and a normal longitudinal ridge (yellow arrow). (**j**) GIIIb with minor irregularity of transverse creases. (**K**) GIVb with multiple depressions and corrugated larva cuticle. (**l**) GVb with a completely dead larva retaining a disfigured cuticle with the appearance of multiple blebs (yellow arrow) and carrions (green arrow). (**m**) GVIb with loss of annulations at the posterior end (blue arrow). (**n**) GVIIb shows a collapsed larva with a loss of normal appearance of the longitudinal ridge with multiple fissures at the cuticle (blue arrow)

Compared to the typical adult in GIIa (Fig. 3a, b), *T. spiralis* adults in GVa and GVIIa attained a severely collapsed body with loss of transverse striations, damaged longitudinal ridges with fissures, and flattened annulations at the posterior end (Fig. 3e, f,h). Adult worm in GIVa showed focal destruction in the epidermis of the wall with blebs, a mild degree of carrion, and loss of transverse striations (Fig. 3d). A disfigurement of the cuticle with multiple depressions, the disappearance of intact annuli with corrugation, and shrinking of the surface epidermal layer were seen in GVIa (Fig. 3g). In GIIIa, the adults showed an intact, smooth, non-wrinkled epidermis with a cauliflower mass appearing on the body and multiple depressions (Fig. 3c).

*T. spiralis* larvae in GVb and GVIIb were dead with a collapsed body and disfigured cuticle in the company of multiple blebs and carrions (Fig. 3l, n) compared to the normal *T. spiralis* larvae in GIIb (Fig. 3i). The larvae in GIVb showed a corrugated cuticle with multiple depressions (Fig. 3k), while in GVIb, loss of annulations at the posterior end was observed (Fig. 3m). However, a minor irregularity of transverse creases was detected in the larvae of GIIIb (Fig. 3j).

## Discussion

Over the past two decades, epidemiological data pertaining to Trichinella spp. infections in people or animals have been documented in 95 countries around the globe. These include 32 countries with a domestic cycle, 75 countries with a wild cycle, and 47 countries with human illnesses [29]. *Trichinella spiralis* is a parasitic worm that goes through three developmental phases in the same host. Because of this, it has been used as a useful experimental model to evaluate the efficacy of various anthelminthic drugs [30]. Albendazole and mebendazole, two benzimidazole derivatives, are the mainstays of therapy for trichinellosis. Nevertheless, treatment with these medications to fight the parasite’s larval stages has dismal results, as reported by Caner et al. [31].

Consequently, one of the main objectives of medical research is to create novel, safe, and effective anthelminthic combinations to combat the encapsulated muscle larvae of *T. spiralis*. Combination treatment aims to enhance therapeutic efficacy and postpone the emergence of resistant parasites by using the synergistic or additive properties of two or more medications. Studies conducted in vivo are more beneficial than those conducted in vitro since many active pharmaceuticals that are active in vitro are inert in living organisms due to a variety of factors, including fast absorption, detoxification, excretion, and metabolism by gut microbiota or liver enzymes [32]. As a result, we reported the findings while carrying out more research on experimental animals to be certain.

For drugs to achieve anti-helminthic efficacy, it is a matter of ultimate importance. This approach seems to result in lower costs, a minor hazard of failure, more safety, and speedier time during the drug development process [33]. Acetazolamide was used in our research as one of the CA inhibitors against *T. spiralis* [8] and atorvastatin as an anti-angiogenicagent [13].

Throughout our investigation, colchicine improved the effects of acetazolamide and atorvastatin on *T. spiralis* adults and larvae. The highest percentages of reductions in adult worms were found in GVa and GVIIa, where they rose from 62.9% in GIVa to 71.2% in GVa and from 35.4% in GVIa to 41.9% in GVIIa. In *T. spiralis* larvae, the percentage reductions rose from 60.3% in GIVb to 75.2% in GVb, and from 33.3% in GVIb to 43.6% in GVIIb. Even though GIIIa and b treated with colchicine resulted in minor percentage reductions in adults and larvae, colchicine had a synergistic effect when coupled with acetazolamide or atorvastatin. Ranjan et al. [34] found that colchicine’s anthelmintic properties are connected to the interaction zones of helminths’ β-tubulin colchicine binding domains. Similarly, Nikolaidis et al. [21] reported that colchicine has an antifibrotic impact by inhibiting fiber construction and deposition. This potential might have far-reaching ramifications for colchicine in other fiber deposition scenarios. As the primary objective of treating trichinellosis is to destroy the encapsulated nurse cell, the use of antifibrotic medications like colchicine as adjuvant therapy has emerged as a highly promising therapeutic approach [19].

Due to their market accessibility, bioavailability, pharmacokinetics, and large therapeutic window, the synergistic combinations of colchicine with either acetazolamide or atorvastatin have several benefits over *T. spiralis*. As well, the two medications’ synergistic action reduces their adverse effects and boosts their biological activity. Additionally, the mixes’ chemical complexity lowers the possibility of drug resistance. This improvement might be attributed to colchicine’s antifibrotic effect, which, according to Hassan et al. [35], either targets the breakdown of nurse cell collagen or prevents it from forming. The increase in the drugs’ efficacy following the addition of colchicine as adjuvant therapy was consistent with the findings of Shoheib et al. [19], who found that the reduction rates of *T. spiralis* larval counts of albendazole alone and albendazole combined with colchicine were approximately 65.2% and 74.9%, respectively. According to Jiang et al. [36], colchicine significantly reduced the amount of collagen fibrils that were deposited around hepatocytes and improved liver cell recovery in schistosomal hepatic fibrosis.

Our findings demonstrated that *T. spiralis*-infected mice had shorter and damaged intestinal villi, which suggested that the intestinal mucosa had also been harmed and that the ability to absorb and digest had been reduced. In contrast to the infected control group, which had finger-like villi and appeared to have intact mucosa, the intestinal sections of the targeted group GVa displayed a notable decrease in histopathological changes, including the inflammatory cellular infiltrate in the small intestine. Hamed et al. [37] approved the significant damage caused by *T. spiralis* infection in the same scenario.

When the mice receiving combined therapy in GV and GVII experienced thinning of the surrounding capsules, marked degeneration of muscle larvae, and replacement of these with amorphous material, the histopathological examination of the infected skeletal and lingual muscles revealed diffuse degenerative changes and inflammatory cellular infiltration that were greatly improved. These effects were more noticeable in the targeted group receiving acetazolamide plus colchicine.

A limited percentage of malformed digested larvae were detected after the single therapy, which may be explained by the fact that the collagen capsule found in nurse cells acts as a barrier against drugs that reduce their effectiveness. Given that the parasite is surrounded by a collagen capsule made up mostly of two kinds of collagen (IV and VI), which supports the parasite’s long-term survival inside its muscle niche [38], Colchicine had inhibitory effects on the expression of genes associated with fibrosis, including collagen-1, collagen-3, α-SMA, and proinflammatory indicators including IL-1β-induced IL-6 [39]. A compromised MT cytoskeleton may be the cause of colchicine’s anti-inflammatory action, as it affects multiple inflammatory pathways in immune-mediating cells. This includes adhesion of neutrophils, activation of the inflammasome, and production of superoxide, in addition to interfering with pathways of RhoA/Rho effector kinase (ROCK) and tumour necrosis factor-alpha (TNF-α)-induced nuclear factor κB (NF-κB), therefore reducing the inflammatory response [40]. Moreover, acetazolamide has anti-inflammatory and anti-oxidant qualities that may aid in enhancing the state of the muscle, according to Beckman et al. [41].

In the current study, the lower adult count was followed histologically by a reduction in inflammatory cellular infiltration in both groups receiving the combination treatment. Similarly, in both targeted groups, a drop in larval count in the muscles was followed by a restoration of muscle fiber architecture and a reduction in inflammatory cells. In the muscular phase of trichinellosis, host tissue damage is induced not only by the invading parasite but also by the presence of inflammatory cells, which create significant quantities of reactive oxygen species and various stress indicators such as transferases and cyclooxygenases [42]. On the other hand, compared to the other groups receiving single therapy, the targeted groups receiving combination therapy experienced less pathological damage to their muscle cells and a decrease in the infiltration of inflammatory cells around the encysted larvae.

Because the main route of drug penetration into helminths is transcuticular passive diffusion, tegumental changes can be considered a useful predictor of a medication’s anthelmintic efficacy [43]. Our SEM results showed that *T. spiralis* adults and larvae treated with colchicine plus either acetazolamide or atorvastatin had collapsed adult worms and dead larvae with substantial tegmental distortion and blebbing. Blebbing is the parasite’s attempt to repair the damaged surface membrane in response to medication activity [44]. However, *T. spiralis* adults and larvae treated with single therapy showed less destructive changes. Although colchicine alone produced minor ultrastructural alterations in *T. spiralis* adults, it caused significant modifications when coupled with acetazolamide or atorvastatin. Interestingly, groups given colchicine and acetazolamide outperformed those given colchicine and atorvastatin.

## Conclusion

Our study demonstrated for the first time that colchicine and either acetazolamide or atorvastatin had a synergistic effect as a treatment characteristic of *T. spiralis* infections. This was confirmed by a statistically significant decrease in mean total adult and larval counts in groups that got combination therapy compared to those that received only anti-parasitic medication. Histopathological studies confirmed these findings by restoring normal intestine and muscle architecture as a result of *T. spiralis* capsule thinning, which increased drug permeability. Moreover, severe ultra-structural changes were observed by SEM.

## Data Availability

No datasets were generated or analysed during the current study.
